# Patients' Preoperative Expectation and Outcome of Cataract Surgery at Jimma University Specialized Hospital -Department of Ophthalmology

**DOI:** 10.4314/ejhs.v21i1.69044

**Published:** 2011-03

**Authors:** Zelalem Addisu, Berhan Solomon

**Affiliations:** 1 Grarbet Hospital Eye Unit, Butajira, Ethiopia; 2 Jimma University, College of Public Health & Medical Sciences, Ophthalmology Department, Jimma, Ethiopia

**Keywords:** Cataract surgery, patient's satisfaction, visual function, Jimma, Ethiopia

## Abstract

**Background:**

Patient's satisfaction for a given treatment is an important clinical outcome because a satisfied patient is more likely to comply with treatments, attend follow-ups and advocate the service to others. Therefore, knowing patients' expectations before a planned procedure or treatment and the actual level of satisfaction and fulfillment of their initial expectations thereafter is much helpful. As far as the knowledge and experience of the researchers is concerned, there has not been any study conducted in Ethiopia to find out about patients' preoperative expectations and postoperative level of satisfaction for actual outcomes. This study was therefore, conducted to describe and find out the relationship between preoperative expectations of cataract patients and the actual postoperative experience and their satisfaction level following the surgery at ophthalmology department in Jimma University Specialized Hospital.

**Methods:**

A prospective cohort study of patients undergoing first eye cataract surgery was conducted from July 10 to Oct., 10, 2007 in the Ophthalmology department of Jimma University Specialized Hospital. Detailed interviews that included general and vision specific health status measures and patients' preoperative expectations for cataract surgery outcomes were performed followed by visual acuity testing. Postoperatively, visual acuity testing was taken again and patients' level of satisfaction with attained postoperative vision was assessed. Data were collected and filled in a separate questionnaire form for each patient, and entered into a computer and analyzed using SPSS for Windows version 12.0.

**Results:**

Of the 200 patients operated for cataract, 179 (89.5%) were followed for the whole five weeks. The average expected preoperative Visual Function-15 score was 96.3, compared to an achieved (postoperative) Visual Function-15 score of just 96.2. However, the most unrealistic expectations observed were reading small prints and doing fine handiwork. The final pinhole visual acuity postoperatively was ≤ 6/18 in 126 (70.4%) patients. Of the 78 (39%) patients who were bilaterally blind preoperatively, 5 (2.5%) patients remained blind postoperatively.

**Conclusions:**

Significant improvements were obtained in clinical, functional, and perceived vision by cataract surgery involving extracapsular cataract extraction with posterior chamber intraocular lens implantations. Expectations regarding visual functioning after cataract surgery were very high, and in most cases and in most cases they were fulfilled.

## Introduction

Cataract is the commonest cause of blindness in the world and the most common forms of it are not still prone to effective prevention and great efforts are therefore being made to provide sight-restoring surgery. Cataract surgery with implantation of an intraocular lens is a sophisticated technological procedure that permits the rehabilitation of vision in the great majority of cases. However, in developing countries, difficulties in accessing eye care both due to individual and environmental factors as well hurdles set up by the health system itself restrict the full utilization of the surgical procedure (1).

Cataract surgery has had a substantial impact in improving the quality of life for individuals with visual compromise secondary to cataract. In recent decades, techniques for cataract extraction have been revolutionized resulting shortening recovery time and increased visual outcome expectations both for the patient and the surgeon.

By the outcome of cataract surgery, we understand the change in functional disability as a result of cataract operation. This can be expressed in different ways: in terms of visual acuity, visual functioning and/or quality of life. It has been increasingly recognized that visual acuity alone may not be sensitive enough to measure the change in functional disability (2).

Patient's satisfaction has become an increasingly important objective for health services providers and professionals which have delivered the reality that the choice and success of many treatments are based on subjective patient-defined criteria (3), as well making patient's satisfaction an element of health status itself. Currently, health care is becoming increasingly privatized and economically competitive with satisfied patients both remaining with and recommending their provider and the best defense against malpractice lawsuits (4).

Assessing visual function is a lengthy procedure requiring experienced interviewers and detailed questionnaires to be filled. In these questionnaires, patients are asked what activities they could do before and after surgery. The questionnaires used cannot be standardized as the activities solicited have to be appropriate for the study population.

The purpose of this longitudinal prospective study was to assess patients' preoperative expectations regarding the outcome of cataract surgery and the actual postoperative experience and to assess the level of satisfaction and evaluate the outcome of cataract surgery at Jimma university Specialized Hospital Ophthalmology department (JUSHOD).

## Materials and Methods

JUSHOD was conducted from July 10, to Oct., 10, 2007. Jimma University Specialized Hospital (JUSH) provides specialized health care services to communities residing in the southwest Ethiopia. Academically, JUSH provides postgraduate trainings in many fields including ophthalmology which started ophthalmic residency and BSc (in cataract surgery) trainings in 2006. JUSHOD serves as a referral eye center for a population of over 10 million residing in southwestern part of Ethiopia as it is the only tertiary eye centre in the area.

The study included all patients aged 12 years and above who underwent cataract surgery from July 10 – Oct. 10, 2007. Patients who were younger than 12 years were excluded as they may be too young to comprehend and properly narrate their preoperative expectations and postoperative outcomes. Hence, all the cases available during the study period and who were eligible were included and available (convenient) sampling was used.

As the data were qualitative and quantitative, structured questionnaire formats were prepared and used to collect the necessary information. During data collection cataract patients were directly interviewed individually by the investigator. After obtaining informed consent, the patient's age, sex, and address were recorded. Preoperative assessment included visual acuity (VA) testing (with Snellen's or illiterate E-charts), intraocular pressure measurements and slit lamp biomicroscopy; keratometry and A-scan biometry were also performed to know the intraocular lens (IOL) power required. Intra-operative and postoperative complications and postoperative VA were measured.

Visual function was measured using a 15-item visual function assessment (VFA) questionnaire (a slightly modified version of the 14-item Visual Functioning Index (VF-14)) (5), a widely used scale based on trouble in conducting common binocular activities, with final score ranging from zero (no visual ability) to 100 (no visual disability). Patients were then asked to rate their preoperative status, expected postoperative functional outcome and the actual outcome for each of the 15 items on the VF-15 scale. Though pinhole VA was taken, not all patients were properly refracted postoperatively (those with borderline and poor VA [VA<6/18] were refracted while those with good VA were not).

The preoperative and postoperative visual status was classified using the World Health Organization (WHO) category of Visual Impairment and Blindness. The standard parameters of assessing quality or outcome of cataract surgery described by Foster and the WHO guidelines for monitoring the outcome of cataract surgery were used (6).

Data collections were conducted before surgery, immediately after surgery, first postoperative day and 5 weeks after surgery as changes in VA are likely to occur within these intervals. The surgical wound usually starts stabilizing from 5th week onwards and the VA at 5–6 weeks postoperatively is more likely the final vision patients attain until late postoperative complications might develop. Finally, the data were entered into computer and analyzed using SPSS version 12.0 for Windows. Spearman rank correlations were used to measure the relationship between overall satisfaction and aspects of expected or achieved visual function (each item of the VF-15 is recorded on a 5-point ordinal scale).

The study was conducted after obtaining ethical approval from Jimma University student research program and the hospital administration. The purpose of the study was explained to the patients under the study and written consent was obtained from each patient (and from parents of patients <18 years old).

## Results

Two hundred patients were approached and requested to take part with all of them agreeing to participate in the study. Of these, 179 (89%) of them were provided followed-up until the 5th week. Out of these 106 (59.2%) of them were males and the others 73 (40.8%) females with a male to female ratio of 1.5:1. Their age ranged from 12 years to 100 years with mean age of 58.3 years and median of 58 years (Table 1).

**Table 1:** Patients' background characteristics, JUDO, 2007.

Characteristics	Preoperative	5 weeks postoperative
Number of patients	200	179 (89.5%)
Mean age (SD)	58.7 (14.6)	58.3 (14.9)
Number of women (%)	80 (40 %)	73 (40.8)
VF-15 score, mean (SD)	54.19 (34.1)	96.2 (11.56)
Overall satisfaction very much improved		132 (73.7%)

The preoperative VA of the designated surgical eye was recorded for all patients. Preoperative best corrected VA of the designated surgical eye was < 3/60 in 190 (95%) of 200 patients and of these, 78 (39%) of the patients were bilaterally blind (VA < 3/60 in the better eye with best correction).Of the 200 planned procedures, extracapsular cataract extraction with posterior chamber intraocular lens (ECCE+PC-IOL) implantation was preformed for 193 (97%) patients. Posterior capsular rupture with vitreous loss occurred in 7 patients of whom 5 (2.5%) patients got anterior chamber IOL (AC-IOL) implantation and the remaining 2 (1%) patients were left aphakic as it was difficult to insert IOL.

**Fig 1:**
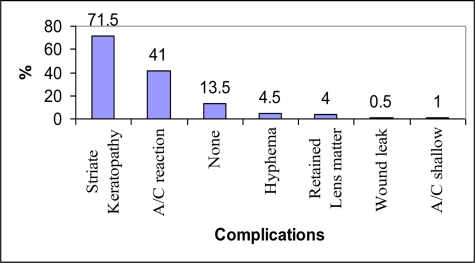
Immediate postoperative surgical events of cataract surgery, JUDO, 2007.

Out of the 200 eyes operated, 182 (91%) were completed without significant intraoperative complications. However, the most frequent complication encountered was posterior capsular rupture which occurred in 10 eyes (5%). And the commonest immediate postoperative complication was striate keratopathy followed by AC reaction, hyphema and retained lens cortical matter while one case of wound gaping required resuturing (Fig 1).

The uncorrected postoperative VA of 102 (57%) patients was 6/18 or better while 77 (43%) patients had VA of less <6/18. However, the final pinhole post-operative VA was 6/18 or better in 126 (70%) patients, and remained <6/18 in 53 (29%) patients (Table 2). A review of 14 patients who did not achieve VA of 6/60 or better at the 5th week showed ocular co-morbidities (age-related macular degeneration (ARMD), pseudo-exfoliation or glaucoma) in 7 (50%) of them.

**Table 2:** Postoperative visual acuity of patients (5 weeks after surgery), JUDO, 2007.

Postoperative VA	Without correction number (%) n=179	With pinhole number (%) n=179
Good (>6/18)	102 (57.0)	126 (70.4)
Borderline (<6/18–6/60)	55 (30.7)	39 (21.8)
Poor (<6/60)	22 (12.3)	14 (7.8)

Of the 200 patients given appointment for follow-up, 179 (90%) patients appeared on the 3rd follow-up visit (5 weeks after surgery) and misshapen pupil (pupil not round) was the commonest postoperative complication at this follow-up time appearing in 21 (12%) eyes followed by visually significant posterior capsular opacity (PCO) which occurred in 9 (5%) eyes (Table 3).

**Table 3:** Postoperative complications at 5th week following cataract surgery in 179 patients, JUDO, 2007.

Postoperative complications	Number	Percent
Misshapen pupil	21	11.7
PCO	9	5.0
Cortical remnants	3	1.7
IOL decenteration	2	1.1
Bullous keratopathy/Corneal opacity/	2	1.1
Uveitis	2	1.1
Wound gape	1	0.6
Pupillary fibrinous material, Vitreous in A/C & iridocorneal touch	1	0.6
Total with complications	41	22.9
None (no complications)	138	77.1

Total	179	100.0

Each item of the VF-15 scale and the mean (±SD) values of patients' preoperative expectations and the postoperative outcome are shown in table 4. The correlation between patients' expectation and the actual outcome is shown in table 5.

**Table 4:** Mean values of patients' preoperative expectation and postoperative outcome for visual function index (VF-15) items, JUDO, 2007.

VF-15 Item	Expectation Mean (SD)	Outcome Mean (SD)	P-value
1. Performing manual tasks?	91(18.6)	96(13.3)	0.12
2. Going out by yourself?	96(10.5)	97(11.9)	< 0.001
3. Reading small print?	93(14.9)	91(18.8)	0.026
4. Reading large print (such as posters), and numbers on telephone?	97(8.9)	97(12.8)	0.7
5. Recognizing people?	97(9.08)	97(12.4)	< 0.001
6. Doing fine handworks like sewing, knitting or wood working?	93(13.4)	88(24.4)	< 0.001
7. Cooking, dressing or other self-care activities?	97(9.96)	96(13.8)	0.07
8. Participating in social activities?	97(8.88)	96(15.0)	0.001
9. Going to shops/markets?	96(10.1)	96(15.7)	< 0.001
10. Working just like you did before?	96(10.8)	96(12.5)	< 0.021
11. Choosing clothes to wear?	99(4.8)	99(3.2)	< 0.001
12. Taking a bath by yourself?	99(4.8)	99(3.2)	< 0.001
13. Getting dressed by yourself?	99(4.3)	99(3.2)	< 0.001
14. Visiting your friends?	98(6.6)	97(13.2)	0.18
15. Watching television?	97(10.9)	100(.00)	0.17

**Table 5:** Sperman correlation with patient satisfaction for composite measures, JUDO, 2007.

*Measure*	*Correlation with satisfaction*	*p-value*
Expected VF-15 score improvement	0.004	NS*
Actual VF-15 score improvement	0.19	NS*
Actual postoperative VF-15 score	0.38	0.01

* NS -not significant

## Discussion

Follow up of patients following surgery is a problem in our department as well in other developing countries' eye units (2). Patients may be forced to travel long distances in order to obtain treatments and hence dropout from consecutive follow-ups is common and the rate increases with longer follow-up periods. This study was having a 5-week follow-up period and 179 (90%) of the 200 patients appeared on the 5th week and this was comparable to similar studies done in other developing countries (2). The percentage of patients who were bilaterally blind preoperatively in this study (39%) is comparable to that of studies made in Sierra Leone (51%) and Nigeria (25%) (7, 8). In our study, 143 eyes were operated by senior ophthalmologists, 12 eyes were operated by residents and the remaining 45 eyes were operated by trainee cataract surgeons under supervision by ophthalmologists.

In this study 38 (19%) eyes were having different ocular co-morbidities with the cataract, namely ARMD, glaucoma, diabetic retinopathy, central corneal opacity, zonular subluxation, uveitis and chorioretinal degeneration. These co-morbidities actually affected the final visual outcome significantly as 50% of those patients whose VA remained <6/60 was having one of these ocular morbidities. Posterior capsular tears were the most frequent major intraoperative complication occurring in 10 (5%) eyes, seven of which had also vitreous loss. Although similar to the incidence in Finland (5.4%) (9), it contrasts with that of Aravind Eye Hospital, India (1.7%) (10). Larger incidences of capsular tears had been reported in Ghana (10%) (6) and Sierra Leone (11.4%) (7). Studies suggest that capsular tears may be more common in Africa than in industrialized countries (8), this may be mainly because of the type of cataract in African patients (dense, hypermature cataracts are common in Africa). Besides, the relative inexperience of surgeons, eye units functioning as a teaching centers and lack of adequate equipments and proper surgical instruments may also play a significant role in the incidence of such complications. Furthermore, Posterior capsule tear was also the main reason in this study for not inserting PC-IOL.

In this study, a higher incidence of immediate postoperative corneal edema (striate keratopathy) (71.5%) than that of the study conducted in Nigeria (44.6%) was observed (8). This difference might be due to the very hard hypermature cataracts in many of our patients plus presence of trainee surgeons new to the technique who need prolonged surgical time with excessive intraoperative manipulations causing endothelial cell loss and corneal edema. The corneal edema however, cleared out almost in all patients in the subsequent follow-up periods.

The most frequent postoperative complication at the 5^th^ week was misshapen pupil followed by PCO. Only eight (4%) eyes developed significant PCO within 5 weeks postoperatively. It has been reported that mature cataracts are at a significantly lower risk for PCO (11). Many of our patients in this study had mature cataracts hence reduced risk of developing PCO. Moreover, our follow-up time was short to reveal all patients who might develop this complication in the long run.

On the other hand, no case of postoperative endophthalmitis was observed within five weeks in our study. Hence this study, as also shown in another study (12), indicated that postoperative infections can be markedly minimized (if not prevented) in developing countries theatre set ups as well though our study was not large enough and follow-up period was also short to get chronic cases of postoperative endophthalmitis.

The postoperative visual outcomes in this study (table 2) were similar to that of a study done in Nepal (13); uncorrected VA of ≥6/18 was obtained in 47.9% of the patients but with correction 77.4% of the patients had had VA of ≥6/18. Our result is also comparable to that of the study conducted at Menelik II Hospital (14) where 42.5% and 57.5% of the patients were having uncorrected VA of ≥6/18 and <6/18 respectively. However, corrected VA was ≥6/18 in 63.2 % of the patients and <6/18 in 36.8 % of the patients. But unlike these studies, in a study done at Kikuyu Eye Unit in Kenya (15), uncorrected VA of ≥6/18 was obtained in 73% of the patients 2 months after surgery.

Based on the WHO guideline for evaluating the outcome of cataract surgery (16), most of our patients (70.4%) had a good outcome and 21.8% had a borderline outcome (table 2). This is much lower than the target set by WHO of 90% or more for a good outcome using available correction. 7.8% of our patients had a poor outcome with VA <6/60 immediately after surgery. This is higher than the target set by WHO of ≥5% for poor outcome. However, VA improved on subsequent follow-ups and further improvement was expected at later follow-up periods. Postoperative refraction 5–6 weeks after cataract extraction has to be done routinely for those with borderline and poor outcome to correct any residual refractive errors. Proper preoperative patient evaluation and identification of ocular co-morbidities also helps to attain the targets set by WHO.

Patients had very high expectations for postoperative function, expecting to achieve a mean (SD) VF-15 score of 96.3 (9.8). Seventy-two patients (36%) expected to achieve a perfect VF-15 score of 100. Only 23 patients did not expect at least some VF-15 improvement, 17 because their preoperative VF-15 score was already 100; the others six all had preoperative VF-15 scores higher than 90 and expected the same VF-15 score postoperatively. As shown in table 4, reading small print(93 Vs 91) doing fine handiwork(93 Vs 88), participating in social activity(97 Vs 96) and visiting their friends(98 Vs 97, are the items for which patients expected the greatest degree of improvement but actual outcomes were slightly furthest from what they expected.

The actual outcome was marginally better correlated than expected improvement and the degree of improvement was not significantly related to patient satisfaction at all (0.38 vs. 0.004) (see table 5). It means that patient satisfaction depends not only on the actual visual outcome they attain but also on the level of their preoperative expectations. Contrary to common expectations, the improvement in visual function experienced by patients did not at all significantly correlate with overall satisfaction. However, expected improvement and actual outcome were all moderately correlated with satisfaction. Clearly patient satisfaction is a complex and multidimensional construct that cannot be explained by single variable like VA improvement (17).

The findings in this study revealed that patients' expectations were high and many of the patients realistically achieved them and also it appears that that ocular co-morbidity is among the major predictors of patient dissatisfaction with cataract surgery (15).

Health care professionals are expected to control their patients' expectations and understanding of treatment to provide the highest level of satisfaction (18). In fact to improve patient satisfaction health care professionals would be advised to pay more attention to patient understanding and expectations even at the expense of improving patient outcome. The close relationship between patient expectations and adequate informed consent cannot be ignored (19). In this study the degree of improvement in visual function was not significantly correlated with patient satisfaction.

This study showed the particular areas where greater focus on asking patients' preoperative expectations would be given. Patients need to understand and be well informed and advised preoperatively about all the possible outcomes of cataract surgery especially when guarded prognosis is anticipated as in cases with ocular co morbidities. Well informed patients about a possible poor outcome may not have very high expectations preoperatively and so unlikely to be disappointed or dissatisfied with whatever final visual outcome they attain.

Our study indicated that perceived patient understanding is an important factor in overall patient satisfaction for a given treatment. It also showed that preoperative understanding and counseling for all possible outcomes (especially when guarded prognosis is anticipated) helps in improving overall patient satisfaction irrespective of the final actual outcome.
